# A Neurocomputational Model of Observation-Based Decision Making with a Focus on Trust †

**DOI:** 10.3390/brainsci16050477

**Published:** 2026-04-29

**Authors:** Azadeh Hassannejad Nazir, Jeanette Hellgren Kotaleski, Hans Liljenström

**Affiliations:** 1 Department of Neuroscience, Karolinska Institute, 17157 Solna, Sweden; jeanette@kth.se; 2 Department of Energy and Technology, Swedish University of Agricultural Sciences (SLU), P.O. Box 7032, 75007 Uppsala, Sweden; hans.liljenstrom@slu.se; 3 Agora for Biosystems, P.O. Box 57, 19322 Sigtuna, Sweden; 4 School of Electrical Engineering and Computer Science, Kungliga Tekniska Högskolan, 11428 Stockholm, Sweden

**Keywords:** neurocomputational modeling, Dynamic Bayesian Probability, emotion, rationality, trust, neurodynamics

## Abstract

**Highlights:**

**What are the main findings?**

- A neurocomputational model linking OFC, LPFC, and ACC explains how observational learning and trust jointly shape decision making.
- Trust dynamically modulates neural oscillatory activity, driving changes in emotional and rational valuation.

**What are the implications of the main findings?**

- The model provides a mechanistic bridge between social learning, neural dynamics, and decision making processes.
- It provides a computational framework for predicting behavior in real-world contexts where decisions are influenced by observing others (e.g., climate-related choices).

**Abstract:**

As social beings, humans make decisions partly based on social interaction. Observing the behavior of others can lead to learning from and about them, potentially increasing trust and prompting trust-based behavioral changes. Observation-based decision making involves different neural structures. The orbitofrontal cortex (OFC) and lateral prefrontal cortex (LPFC) are known as neural structures mainly involved in processing emotional and cognitive decision values, respectively, while the anterior cingulate cortex (ACC) plays a pivotal role as a social hub, integrating the afferent expectancy signals from the OFC and LPFC. This paper presents a neurocomputational model of the interplay between observational learning and trust, as well as their role in individual decision making. Hence, our model provides a framework for investigating how emotional and rational responses may change when individuals observe the action–outcome associations of an alleged expert. We have modeled the neurodynamics of three cortical structures (OFC, LPFC, and ACC) and their interactions, where the neural oscillatory properties, modeled with Dynamic Bayesian Probability, represent the observer’s attitude towards the expert and the decision options. As an example of an everyday behavioral situation related to climate change, we use the choice of transportation between home and work. The model generates EEG-like signals that show how patterns of neural activity change during observation-based decision making. The simulations suggest that higher levels of trust influence both emotional and rational evaluations when individuals observe the actions and outcomes of an expert. Overall, the proposed framework provides insight into how observational learning and trust work together to shape decision making. It highlights the dynamic interplay between emotional and cognitive processes and offers a mechanistic understanding of how social information can influence behavior.

## 1. Introduction

Observational learning plays an important role in many forms of human decision making, particularly in situations where individuals rely on the experiences and outcomes of others to guide their own choices. In such contexts, trust in the observed agent and the evaluation of observed outcomes influence whether individuals adopt or reject a particular strategy. Understanding these processes requires integrating insights from both social processes and the neural systems that support learning and valuation. This study therefore considers observational learning and trust-based decision making from both social and neural viewpoints, providing a framework that links behavioral processes with their possible neural substrates. Reviewing these perspectives provides a basis for introducing a neurocomputational framework that links these two levels of analysis through a computational model.

### 1.1. Background

To provide the necessary context for the present study, we first review relevant perspectives on observational learning and trust-based decision making at both the social and neural levels.

#### 1.1.1. Social-Based Decision Making

Human decisions are influenced by information from various sources in the environment. Decision making and learning are two concepts strongly linked together, with each serving as an inseparable basis for the other [1,2,3,4]. The goal of human decision making could be considered maximizing its utility [5,6], where different types of contextual learning (i.e., individual and social context) contribute to the knowledge base for reaching a decision [7,8].

While an individual’s own experiences may be the primary reason behind his/her learning, learning from others, i.e., social learning, also plays an important role [9]. According to Bandura [10], social learning is based on observation, imitation, and cognitive modeling. In this context, individual learning by observing the behavior of others is known as *observational learning* [11]. Bandura [12] demonstrates different forms of observational learning. Encoding the observed behavior through copying, regardless of the potential consequence, is one form of observational learning. Another form is *observational associative learning*, when the behavioral learning could result from associating an observed action with its outcome. Allowing for a conditional association between the observed action and the outcome, observational learning is the corollary of the generation of prediction error (PE) signals. Two forms of vicarious prediction error, observational action prediction error and observational outcome prediction error, defined as the difference between the anticipated and actual value of others’ behaviors and their outcomes, can account for human observational learning. The sign and magnitude of these signals determine the motivations for engaging in (i.e., rewarding) or withdrawing from (i.e., aversive) a specific behavior [13,14,15,16,17,18,19,20].

The concept of associative learning is strongly related to the concept of goal-directed behavior [21]. According to Dickinson and Balleine [22,23], goal-directed behavior is based on the contingency of association between action and outcome, with the outcome serving as the goal. The motivation behind goal-directed behavior at the individual level is to attain a more desirable outcome. The same logic can be applied to individual learning in a social context. Observing the goal-directed behavior of others may motivate the observer to follow the observed action, in case the outcome is expected to be rewarding. An individual is motivated to increase the probability of their own potential reward by correctly following the observed action.

The conceptual connection between goal-directed behavior and learning can be generalized to the concept of *trust*. Trust, as a social capital, has been defined with various connotations. However, the common issue of ‘expectation’ emerges as a key determinant of trust across all definitions. An individual’s expectations are tied to his/her predictions. Actions and outcome predictions play pivotal roles in building or destroying interpersonal trust [24]. Thus, the issue of predictability concerns the likelihood of action and outcome occurrence [25]. The constancy of a person’s behavior over time, referred to as consistency, can indicate action predictability. On the other hand, the ability of an individual to successfully execute actions, known as competence, determines outcome predictability [26,27].

The behavior or attitude of a person is deemed trustworthy by the observer if following that specific behavior or attitude proves rewarding to the observer. Hence, learning about the action-outcome contingencies through observational associative learning provides a basis for learning about (emerging trust) and from others (behavioral change). Learning about others may lead the observer to make a decision about forming a trusting relationship with them. This process might result in either trust or distrust towards them, indicating the observer’s future actions [28,29,30,31,32]. In this regard, trust has an undeniable impact on social learning and decision making. Trust can improve the effectiveness of decisions made [33], based on what has been learned through observation, here referred to as *observation-based decision making*.

The contributions of different underlying factors, emotion and rationality, in the decision making process are tenable in a trust-based observational learning context [34,35]. The neural activities of distinct emotional and rational structures, proposed by Kahneman [36], underlie the decision making process. These activities have been recorded during an individual’s cognitive behaviors in a social context [37,38]. In the following, we describe the major underlying neural structures of the decision making process in a social context.

The processes described above characterize observational learning and trust-based decision making at the behavioral and social level. However, these processes must ultimately be supported by neural systems that encode value, evaluate observed outcomes, and integrate information relevant for decision making. Understanding how such behavioral phenomena arise therefore requires considering the neural mechanisms that may implement these functions. In the following section, we review candidate cortical systems that have been proposed to support valuation and decision processes involved in observational learning.

#### 1.1.2. The Neural Basis of Social-Based Decision Making

Behavioral processes involved in observational learning and trust-based decision making are thought to rely on neural systems responsible for valuation, prediction, and decision integration. Research in cognitive neuroscience has identified several cortical regions that contribute to these functions, particularly within the prefrontal cortex. These regions have been associated with evaluating observed outcomes, representing expected value, and integrating emotional and cognitive aspects of decision making.

In an earlier study, we presented a neurocomputational model illustrating the involvement of the orbitofrontal cortex (OFC) and lateral prefrontal cortex (LPFC) in the human experience-based decision making process [39]. OFC and LPFC are also considered to be involved in processing social inputs. However, assessing the integrated social inputs requires yet another neural structure, namely the anterior cingulate cortex (ACC).

OFC has long been known as a structure involved in modulating emotional arousal [40,41]. The critical location of this structure profoundly affects its contribution as the recipient of internal efferents from subcortical structures. It contrasts with its role as an integrator of external information from associative sensory structures (i.e., late sensory stimuli).

The strong bidirectional connections of the OFC with the amygdala are pivotal to emotional behavior control. The recorded oscillatory activity of the OFC represents the expected value of the associated outcome of the afferent stimulus, which is called the expectancy signal. OFC puts a high value on the immediate gratification of the associated outcome. Hence, the neural dynamics of this structure are indicative of an individual’s emotional motivation.

The executive functionality of the cortical laminar structure of the LPFC was demonstrated by Pribram and others. The intra-cortical connectivity of the dorsolateral prefrontal cortex serves as an early sensory stimuli processor for subsequent cognitive reasoning [42,43,44]. The oscillatory activity of the LPFC can be represented by the magnitude, time of delivery, and the probability of outcome occurrence of the signal. In contrast to the OFC, the higher valuation of long-term rewards in the LPFC shows the rational aspect of the stimuli. However, both structures pursue goal-directed behaviors [45,46]. The episodic control of neurons located in the mid-LPFC provides a basis for retrieving maintained episodic memory and directing behavior towards a goal [47,48]. In addition, mirror-like neurons were found in the LPFC [49], indicating that it plays a pivotal role in imitating the observed behaviors of others.

The efferent neurons branching out from the LPFC take control of emotional activity by modifying the oscillatory activity of the OFC. The power of the afferent neurons to the OFC affects the integrated emotional and rational values [50,51,52,53,54,55]. Therefore, the final individual decision is the result of an integration of the expectancy signal projected from the OFC and the rational valuation signal from the LPFC.

Burke et al. [13] illustrate that the oscillatory activities of the ventromedial prefrontal cortex (vmPFC) and dorsolateral prefrontal cortex are determinants of the observational action and outcome prediction errors, respectively. Furthermore, the involvement of OFC in predicting social cues has been recorded by Campbell-Meiklejohn et al. [9]. Given the structural and functional overlap between the vmPFC and OFC, these regions may be considered functionally comparable in the context of social decision making. In this regard, the vmPFC/OFC and LPFC not only underlie emotional/rational individual decision making but also serve as bases for observational action-outcome associative learning The integration of social information projected from these structures occurs in the ACC, which is part of the limbic system. According to documented experiments [56,57,58,59], the dorsal part of the ACC takes over the rational activities, while the ventral part is responsible for emotional computations. The laminar distributed pattern of the ACC, similar to that of the LPFC and OFC, comprises the distribution of interneurons and normal pyramid and spindle cells [60,61].

**Figure 1:**
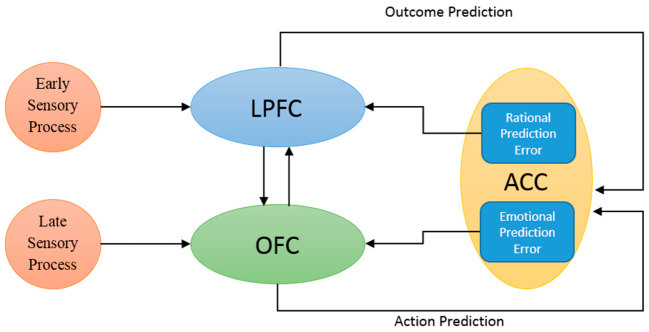
Illustration of the schematic flow of information among three neural structures involved in observational learning. The LPFC and OFC are, respectively, subjected to early rational and late emotional sensory stimuli, projecting the processed input to the ACC. The differences between the actual and predicted observed action and outcome are signaled as emotional and rational prediction errors, resulting in the update of the oscillatory properties of the LPFC and OFC.

The contribution of the ACC to both rational and emotional aspects of human behavior makes it a hub for cortico-cortical and cortico-limbic connections. Hence, ACC is regarded as a hub for social valuation. The afferent and efferent neurons connect different neural structures, including the LPFC and OFC, to the ACC. These connections facilitate the flow of social information within these structures. This attributed function is the result of generating prediction error signals that project from the mentioned structures [62,63,64,65]. The oscillatory activities of the LPFC and OFC are monitored and updated through the modulatory mechanism of ACC, based on a reinforcement learning process [30,66,67]. The schematic diagram illustrating the flow of information in a social context is shown in Figure 1.

Taken together, these findings suggest a correspondence between behavioral processes involved in observational learning and the neural systems that support them. This observation motivates the need for a more explicit framework linking these levels.

#### 1.1.3. Linking Social and Neural Levels

While the above perspectives describe social and neural processes separately, an important step is to clarify how these levels relate to one another within a unified framework. In the present study, social constructs such as trust, observational learning, and decision making are not treated as independent descriptive entities but are considered to emerge from underlying neural processes involved in valuation, prediction, and integration of information.

In this view, emotional and cognitive aspects of behavior correspond to distinct but interacting neural systems, primarily associated with orbitofrontal and lateral prefrontal regions, respectively. The integration of these processes, together with the evaluation of observed actions and outcomes, provides a basis for understanding how social information can influence individual decisions.

The present model integrates these perspectives by introducing trust as a learning-dependent modulation factor, embedding it within a neurocomputational architecture involving OFC, LPFC, and ACC, and linking emotional and rational valuation through oscillatory neural dynamics. In doing so, the model seeks to connect computational principles with neural mechanisms and social processes within a unified framework.

This conceptual linkage serves as the foundation for the neurocomputational model introduced in the following section.

### 1.2. Objectives of This Study

Social interactions exert a significant influence on an individual’s attitudes, thereby defining their behaviors and decisions within a social context. While behavioral change may not always stem directly from modifications to the value system, an individual’s attitude could indeed shift. This occurs in response to the motivational value associated with an action.

The level of interpersonal trust is closely linked to societal influences. The behavior of individuals can be influenced or altered when they observe the actions and outcomes of others. This is determined by the level of trust between the observer and the individual whose actions they are witnessing. The concept outlined above forms the conceptual framework of this project. At its core, this study seeks to investigate potential changes in individual behavior when observing an action and its outcome performed by an individual considered skilled (“expert”). This conceptual foundation not only enables an examination of observational learning but also considers two significant factors: trust and its influence.

In this paper, we explore the reciprocal effects between observational learning and trust. Additionally, we examine how this interaction influences an individual’s emotional and rational assessments, as well as his/her ultimate decision making.

With our neurocomputational model of the three neural structures, OFC, LPFC, and ACC, we have previously explored the oscillatory neurodynamics activity associated with decision making [39]. Additionally, our model explores the behavioral pattern of an observer in a trust-dependent, observation-based decision making process. It relies on the oscillatory activities of the three neural structures. The neural pattern properties demonstrate the values of the corresponding decision options and the unidirectional trust relationship between the observer and the observed expert. We can also illustrate the relationship between changes in trust level and changes in emotional/rational attitude towards the decision options. As a measure of the trust level and its influence on observational learning, we consider the predictability of the action and its outcome. The coding of the contingency between action and outcome provides a basis for individuals to learn about and from others in society. The projected action and outcome prediction error signals update and restore the emotional and rational memory about the consistency and competency of the observed expert, respectively. Thus, the observer’s information about the observed expert will be improved. Furthermore, the neural activities involved in associative learning in a social context provide a basis for learning from society. This is achieved through making associations between the observed action and its outcome [68,69].

Taking into account the variety of decisions we make in our everyday lives [70], the impact of our decisions related to climate change is of special interest here. A central problem, in this regard, is how individual attitudes and associated behavioral patterns can change over time. This is particularly relevant in areas such as travel and transport, which may have a significant impact on climate change.

In this study, we use transportation choice (e.g., car, bicycle, or public transport) as a simple illustrative example of climate-relevant behavior. In everyday life, individuals may notice that others around them are changing their habits or discussing alternative choices. However, rather than modeling diffuse social influence at the population level, the present framework focuses on observational learning from a specific individual.

The purpose of this example is to provide a clear and controlled setting in which the role of trust in observational learning and its influence on decision making can be examined. The scenario is not intended to represent a specific real-world application but rather to illustrate the qualitative behavior of the proposed model.

In this example, the observed “expert” corresponds to the specific agent described above, whose actions and outcomes are tracked over time and evaluated in terms of consistency and predictability.

While such observations occur within a broader social environment, the model focuses on how trust is updated through repeated observation of a single agent.

In everyday situations, these observations may involve a wide range of individuals, from strangers to familiar or trusted persons such as family members or public figures. Within this framework, we explore how emotional and rational values associated with different transport options are influenced by trust-dependent observational learning. The characteristics of both the observer and the observed individual shape the quality of this trust relationship. Consequently, the study is based on specific behavioral and neural assumptions, which are described in the following sections.

## 2. Theory and Models

The primary objective of our study is to illustrate how changes in the oscillatory neurodynamics serve as a hallmark of trust-dependent observational learning, impacting the decision making process. Our goal is to demonstrate the transition of neural activities from heuristic to more rational reasoning, driven by an increase in rational trust.

We hypothesize that the model-generated oscillatory activity of the LPFC is responsible for measuring the level of rational trust. We evaluate the value of trust by measuring the excitability of neural units, simulating an individual’s attitude towards the observed expert. Our neurocomputational model focuses on trust by evaluating the contingencies of the expert’s action-outcome to determine the impact of these variables on (1) the level of trust and (2) the observer’s decision.

### 2.1. Neurocomputational Approach

Our model consists of two parts that focus on the I. individual and II. observation-based decision making process. The first part focuses on the neural units’ activity, encoding anticipated emotional and rational values of the options. The aim is to study the changes in the observer’s neural behaviors influenced by others. This step is a feed-forward decision process with no immediate feedback. The emotional and rational priorities of the observer, as well as the observation of the expert’s action-outcome association, influence the final decision. It is noteworthy that the observer’s predisposition towards the expert’s profession may bias perceptions. This bias potentially leads to positive or negative reevaluation of the expert’s actions, irrespective of their consistency or competence level.

The focus of the second part lies in the neural oscillation involved in vicarious decision making, considering both the observed action and its subsequent outcome. This process relies on two main factors: 1. Predictions of observed actions and 2. Predictions of observed outcomes, which are generated in the OFC and LPFC, respectively. Discrepancies between the observed (actual) action/outcome and the predicted ones drive changes in the neural properties of the structures associated with both the observed expert and the options chosen by them.

**Figure 2:**
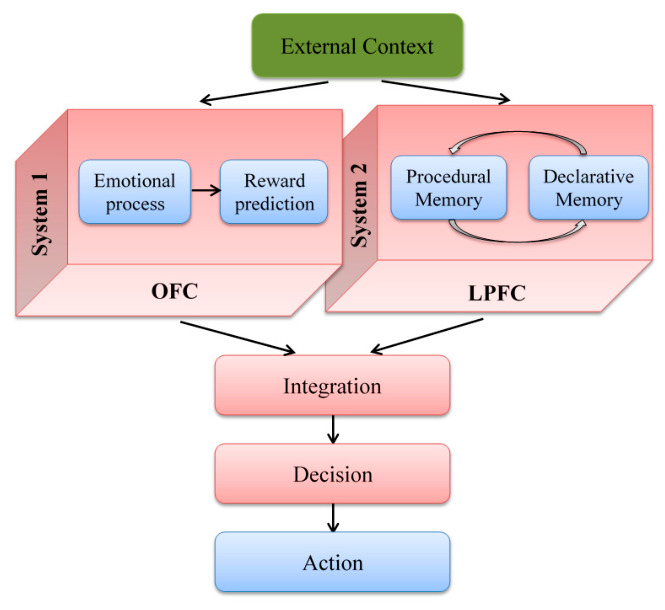
Illustrates individual decision making in the absence of individual experience. Adopted from Hassannejad Nazir and Liljenström [39].

There are different cognitive processes involved in the described process. Mirroring, interactive learning, habitual, and goal-directed valuations are just a few aspects of social learning and decision making processes. These cognitive processes are underpinned by numerous neural structures. In light of an extensive exploration of this topic, we have focused on modeling two specific neural structures involved in emotional, goal-directed behavior and decision making. The orbitofrontal cortex (OFC) primarily handles the encoding of the first two cognitive aspects, while the lateral prefrontal cortex (LPFC) is implicated in the latter two functions. In the social context, the necessity of a coordinating structure that contributes to integrating, monitoring, and controlling socially driven stimuli is evident. Therefore, we focus on simulating activity in the anterior cingulate cortex (ACC). We have developed a neurocomputational model that simulates the decision making process in a social context based on the comparison between prediction and observation of goal-directed behavior. The initial step bears significant resemblance to the neurocomputational model previously developed by Hassannejad Nazir and Liljenström [39], shown in Figure 2.

An experience-based decision stems from comparing the anticipated and actual actions encountered by an individual. Conversely, social-based decision making involves vicarious learning, wherein individuals make decisions based on insights gained from others’ experiences. In our model, the individual decision making process unfolds in the absence of the individual’s direct experiences. As depicted in Figure 2, the initial part of the individual decision making process is driven by feedforward neural activities, with the individual’s own experiences and feedback being overlooked.

**Figure 3:**
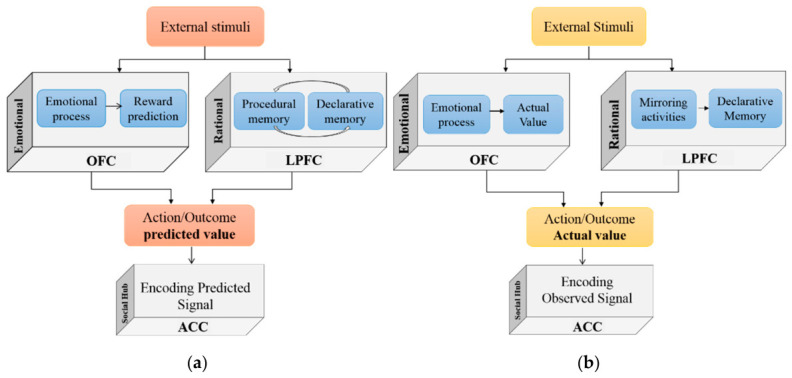
Illustrates (**a**) the schematic demonstration of observational action/outcome prediction and (**b**) action/outcome observation from a neural viewpoint. The emotional and rational value of the observed action is computed vicariously in the OFC and LPFC, respectively. The final value is the result of signal integration, which is projected into the ACC for further processing.

Figure 3 depicts the schematic demonstration of the second part of the model. Here, the OFC and LPFC are responsible for encoding the anticipated and actual emotional and rational values of both the observed expert’s decisions and the vicarious decisions. In LPFC, the neural units signify the rational value of an individual’s decision in the initial stage of the model. Additionally, the involvement of mirror-like neurons in this structure plays a crucial role in assessing the rational value of the observed action-outcome association. The emotional value of the observed action is encoded by OFC, representing its desirability. The two components of the model are intrinsically coupled through shared neural and learning dynamics. The feedforward process establishes the initial emotional and rational value representations within OFC and LPFC, which determine the predicted action–outcome values associated with each option. The simulated orbitofrontal and lateral neurons transmit the anticipated (Figure 3a) and actual (Figure 3b) values (both emotional and rational) of the expert’s action-outcome association to ACC. The integration of these simulated signals in ACC generates errors in observational action and outcome prediction. The model-generated predicted and actual values of the expert’s decisions are influenced by the individual’s emotional-rational experiences, priorities, and prior bias towards the expert.

**Figure 4:**
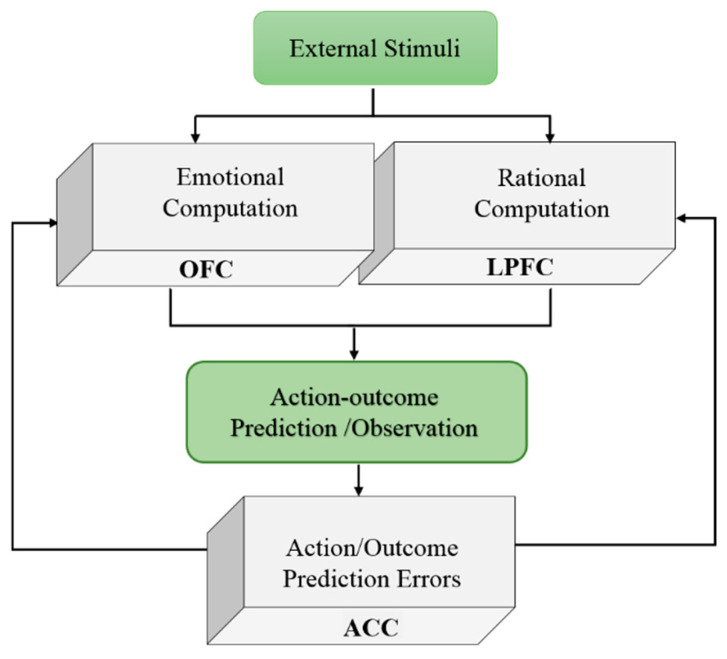
Integration of predicted and actual values. The difference between the values of predicted and actual afferent signals from the OFC and LPFC generates an error-related negativity signal. This signal is then sent back to the emotional and rational structures to update the neural variables such as learning rate, oscillatory properties, and excitability of neural units.

The encoded observational action and outcome prediction errors in the ACC are projected to the OFC and LPFC to update the properties of neural patterns associated with the observed action and the expert, as depicted in Figure 4.

#### 2.1.1. Neural Structures

Despite the variations observed in the structures of the OFC, LPFC, and ACC, all these structures are organized in layers. Taking this into account, Hassannejad Nazir and Liljenström [39] modeled the neurodynamics of the OFC and LPFC based on a three-layered neural model developed by Liljenström [71].

**Figure 5:**
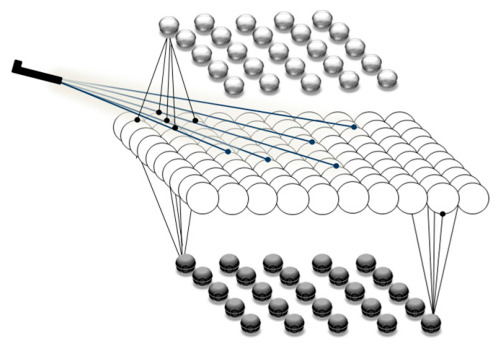
Schematic illustration of a simplified three-layered neural structure consisting of excitatory and inhibitory neural units. The excitatory layer located in the middle is composed of 100 neural units, with two feedforward and feedback inhibitory layers at the top and bottom, respectively, each containing 25 neuronal units.

Considering the neuroanatomy of the ACC, the structure is designed similarly to the OFC and LPFC to avoid the complexity caused by the diversity of cellular composition in the ACC. The upper and lower levels of the three-layered structure comprise inhibitory neurons corresponding to layers III and Vb of the ACC, while the middle layer represents the high density of excitatory cells in layer V of the ACC with excitatory neural units. Hence, the three-layered structure is common among all three structures. This structure is suggested based on the attractor neural network, which provides a basis to generate the oscillatory activities of neural patterns. The modeled activity of cell assemblies represents the processes of memory formation, retrieval, valuation, and prediction. The neural interactions between the middle excitatory neurons and the two feedforward and feedback inhibitory layers, stimulated by external stimuli, are illustrated in Figure 5. Although the structures of these neural parts are notably simplified, the accuracy of their performance has been considered in the developed model.

The oscillatory activities of the neural units have been computed as follows:

(1) $$du_{i}/dt=\;-u_{i}/\tau_{i}+\;\sum_{j\neq i}^{N}\;w_{ij}g_{j}\left(u_{j}\;(t-\delta_{ij})\right)+\;I_{i}\left(t\right)+\;\xi \;(t)$$

where *u_i_* represents the internal states of the neural unit stimulated by the external input, $I_{i}\left(t\right)$. In the model, each element $w_{ij}$ denotes the strength of the connection from unit *j* to unit *i*, with a conduction delay, $\delta_{ij}$*_j_*, and the membrane time constant, *τ_i_*, which play a role in measuring the rate of neural activity changes.

The input-output function, *g_i_*(*u_i_*), measured by Freeman is based on the gain parameter, *Q_i_*, denoting the level of arousal, or the excitability of units to form neural patterns [72]. C is a normalization constant.

(2) $$g_{i}\left(u_{i}\right)=CQ_{i}\left(1-\exp\left[-\exp\left(u_{i}\right)/Q_{i}\right]\right)$$

Here, the learning process follows the Hebbian theory [73]:

(3) $${∆w}_{ij}=\;\alpha g_{i}g_{j}(w_{max}-w_{ij})$$

where α, is the learning rate. The parameter $w_{max}$ represents the maximum allowable connection strength between neural units in the model. It constrains the range of synaptic weights and ensures that the strength of interactions between neural populations remains within biologically plausible limits. The impact of trust on social processes has been considered in the literature. Social learning is an important constituent of social processes, also influenced by trust. The introduction of trust in the learning rule is motivated by prior findings showing that learning dynamics are context-dependent. Hence, to consider the correlation between neural learning and trust, we modified the Hebbian learning equation as follows:

(4) $${∆w}_{ij}=\;\alpha g_{i}g_{j}(w_{max}-\beta .w_{ij})$$

where β implements the modulatory role of trust as defined above. β is defined in the normalized range [0, 1], where 0 corresponds to absence of trust and 1 to maximal trust. The more consistent and competent the observed expert is, the higher the β value will be. The coefficiet β takes a higher value when the observer’s trust in the expert changes.

In this formulation, β does not directly scale the learning rate. It instead affects the effective saturation term of synaptic weights. As a result, changes in β indirectly influence the emergent neural dynamics, including oscillatory activity and signal intensity, during observational learning. An increase in β affects how synaptic weights saturate, leading to more stable learned associations. Specifically, higher values of β lead to earlier saturation of synaptic updates, shaping how strongly and how quickly associations stabilize over time.

The incorporation of trust as a modulation factor in the learning rule is intended as a computational abstraction of the influence of social and contextual variables on learning dynamics. Previous work in neuroscience and reinforcement learning has demonstrated that learning rates and synaptic plasticity are not fixed but are modulated by factors such as uncertainty, reward expectation, and environmental context [74,75].

In this sense, trust can be interpreted as a higher-level variable reflecting the perceived reliability of an observed agent, thereby influencing the strength and rate of associative learning.

We consider the value of each decision option, $V\left(opt\right)$, to be proportional to the weight strength, $W_{opt}$, and energy of the corresponding cell assembly, $E_{opt}$. The energy of the cell assembly is based on the signal’s frequency, $f_{opt}$, and amplitude, $A_{opt}$.:

(5) $$V\left(opt\right)=\;\left|W_{opt}\right|\cdot \;E_{opt}\left(f_{opt},A_{opt}\right),\;\;\;\;\;\forall \;opt=1,\ldots ,n$$

In the present model, trust is defined as a unified computational variable with two complementary roles. First, it functions as a state variable representing the observer’s belief about the reliability and predictability of the observed agent. Second, it acts as a modulator of learning by influencing synaptic plasticity through its incorporation in the modified Hebbian learning rule (Equation (4)).

Importantly, variables such as neural gain (*Q*), connection weights, oscillatory excitability, and dominant frequency do not independently constitute trust. Rather, they represent downstream manifestations of the learning dynamics modulated by trust. In this framework, β influences synaptic updates, which in turn shape connection weights and subsequently give rise to changes in neural excitability and oscillatory activity. β is treated as a unified computational variable, whose effects are expressed through neural dynamics rather than redundantly encoded across multiple parameters.

This establishes a causal and non-redundant mapping in which trust is uniquely encoded by β, while the other variables reflect its dynamical consequences at the neural level, rather than alternative or interchangeable representations.

#### 2.1.2. Neural Aspect of Probability Theory

Understanding how the brain represents uncertainty and encodes probabilistic information is a central question in computational neuroscience. A common approach has been to describe neural activity within a Bayesian framework, where beliefs are continuously updated based on prior knowledge and incoming observations [76,77,78,79,80]. From this perspective, predictions about observed actions and their outcomes are closely intertwined and gradually emerge through experience.

Within this framework, these predictions are not formed independently of the learning process; rather, they emerge from previously observed action–outcome associations and are continuously shaped by trust-dependent modulation. In this sense, the likelihood of an action and its associated outcome reflects not only patterns learned from past observations but also the perceived reliability of the observed agent.

Unlike conventional formulations where learning is driven solely by prediction errors or probabilistic inference, the present model introduces variable β as an explicit, dynamic modulation variable. Trust is updated based on the predictability of observed actions and outcomes and directly influences synaptic plasticity through a modified Hebbian learning rule. This results in a learning process in which prediction errors are measured and weighted by the perceived reliability of the observed agent.

In this regard, it is important to consider how observations evolve over time. For this reason, we use Dynamic Bayesian Probability (DBP) [81] which extends probabilistic inference to sequences rather than isolated events. In simple terms, DBP estimates the likelihood of a future action based on the history of past observations. The following equation indicates the probability of the occurrence of an action at time *n* + 1, $X_{n+1}$, given the past actions based on DBP.

(6) $$\begin{array}{cc} \mathrm{Pr}(X_{n+1}=x|X_{1} & =x_{1},X_{2}=x_{2},\ldots ,\;X_{n}=x_{n})\\ & =\mathrm{Pr}\left(X_{n+1}=x|\;X_{n}=x_{n}\right)\times \mathrm{Pr}\left(X_{n}=x|\;X_{n-1}=x_{n-1}\right)\times \ldots \times \mathrm{Pr}\left(X_{2}=x|\;X_{1}=x_{1}\right) \end{array}$$

This method can be applied for active learning problems while environmental changes are important. Given the history-dependent nature of decision making, the likelihood of the expert making a decision hinges on the associative prediction of past actions and outcomes. The overall approach and attitude of the expert can be inferred through a sequential dependent probability distribution. Therefore, DBP is applied here to regulate the neural oscillatory activities during observations. The strength of the predictive signals (i.e., mean peak value and frequency) is computed based on the probabilities of observed actions and outcomes.

(7) $$\mathrm{DBP}=\mathrm{Pr}\left({AO}_{n+1}=r|{AO}_{1}=r_{1},{AO}_{2}=r_{2},\ldots ,\;{AO}_{n}=r_{n}\right)$$

*AO* represents the action-outcome association, while $r_{n}$ denotes the desired rewarding outcome at time *n*.

The proposed framework relates to well-established computational ideas. It shares features with reinforcement learning, where prediction errors are used to update internal representations, and with predictive coding, where the system continuously minimizes the mismatch between expected and incoming signals. What distinguishes the present model is the explicit role of trust, which modulates how strongly new information influences learning over time.

Here, the emerged prediction errors are accumulated over the duration of each trial and update trust via the DBP formulation. The updated trust, in turn, modulates synaptic plasticity through the modified Hebbian learning rule, thereby influencing the evolution of connection weights and the subsequent neural activity. In this way, neural dynamics, prediction error signals, and trust form a closed-loop system in which the feedforward and observation-based processes are continuously integrated over time.

In this study, the neural coding of probability is measured based on the neural excitability, strength of neural unit connections, and associative connections’ strength. Additionally, we apply the computed probability, *DBP*, to measure the properties of neuronal units encoding uncertainty. Signal intensity is determined by signal energy ($E_{s}$), dynamic probability (*DBP*), and motivation to follow the observed individual ($Q_{indiviudal}$), i.e., trust in the observed individual.

(8) $$Signal\;value=E_{s}\times Q_{individual}\times DBP$$

The signal value represents the magnitude of the prediction signals, indicating the action and outcome predictability of the expert, as well as the probability that the observer follows the expert.

#### 2.1.3. Simulation Design and Reproducibility

The simulations and numerical implementation of the model were performed using *MATLAB R2023a* (MathWorks, Natick, MA, USA). The model is developed based on two interacting time scales: a micro-scale that generates neural dynamics within each trial and a macro-scale that describes how learning unfolds across trials.

##### Micro-Scale (Within-Trial Dynamics)

At the micro-scale, neural activity evolves continuously within each trial, driven by synaptic inputs and network connectivity. Although the dynamics are formulated in continuous time (Equation (1)), they are implemented numerically using a discrete-time approximation with a fixed time step (Δt ≈ 1 ms) and a forward Euler integration scheme. At each time step, the state of each neural unit is updated as follows:

(9) $$u_{i}\;\left(t+∆t\right)=u_{i}\;\left(t\right)+∆t.\frac{du_{i}}{dt}$$

where $\frac{du_{i}}{dt}$ is defined by Equation (1). Synaptic weights, gain parameters, and trust are updated at the trial level based on the accumulated neural activity within each trial. This choice of time step provides a good balance between the temporal dynamics of the neural activity and maintaining computational efficiency.

During each trial, these iterative updates give rise to evolving neural activity patterns from which predicted and observed action–outcome values are measured. These values are compared within the anterior cingulate cortex (ACC), generating prediction error signals.

At each discrete time step within a trial, the model evolves through a sequence of coupled updates that integrate neural dynamics, learning, and trust modulation. Neural activity within each cortical unit is first updated based on the current inputs and synaptic connectivity, as defined by the dynamical equations of the system. The resulting neural activity is accumulated over the duration of the trial and serves as the basis for learning at the macro-scale.

##### Macro-Scale (Across-Trial Learning)

At the macro-scale, the model evolves across discrete trials representing sequential observations of the expert. Each trial corresponds to one instance of observation and learning.

After each trial, the accumulated neural activity is used to update the trust variable β, reflecting the perceived reliability of the observed agent. This update is governed by a Dynamic Bayesian Probability of observed associations. The updated trust then modulates synaptic plasticity, and connection weights are adjusted according to the trust-modulated Hebbian learning rule (Equation (4)).

The full simulation consists of multiple trials (typically 40–100), allowing the model to capture the gradual evolution of trust and decision behavior over time.

##### Coupling Between Time Scales

The two time scales are coupled through a closed-loop interaction. Micro-scale neural dynamics generate prediction errors within each trial, which drive updates in trust at the macro-scale. In turn, the updated trust modulates synaptic plasticity, thereby shaping the evolution of neural dynamics in subsequent trials. This bidirectional interaction enables the co-evolution of neural activity and belief updating within a unified computational framework.

## 3. Simulation and Results

### 3.1. Assumptions

In this study, the conceptual model is based on simulated observational prediction errors related to actions and outcomes within prefrontal cortex regions. The emerging results are not intended as empirical validation, nor do they provide direct predictions of neural activity. Instead, they illustrate how the interaction between observational learning and trust, as defined within the model, can give rise to coherent patterns of neural dynamics and behavioral change.

Therefore, the findings should be understood as qualitative demonstrations of the model’s dynamics rather than definitive empirical claims.

The conceptual models and the applied cortical model are illustrated in Figure 1, Figure 4 and Figure 5. The influences of social information on adaptive heuristics and the rationality of human decision making are simulated and analyzed with respect to some initial behavioral and neural assumptions.

At the start of each simulation, neural states, synaptic weights, gain parameters, and trust values are initialized as follows.

Synaptic weights $w_{ij}$ are initialized using random values drawn from a Gaussian distribution within the interval [0, 1], with their spatial distribution constrained by distance-dependent connectivity between neural units. This ensures biologically plausible initial connectivity while allowing sufficient variability for learning. All weights are bounded above by $w_{max}$ to guarantee stable convergence during learning.

The observer’s decision making is assumed to be shaped by both emotional and rational processes. To investigate the transition between these modes, the observer is initially biased toward emotional processing. Accordingly, the intensity of emotional signals, motivation (*Q*), and neural excitability are set higher than their rational counterparts. The initial parameter values, including *Q*, are summarized in Table 1 together with the variables used in Equations (1)–(4).

In addition to the observer’s characteristics, the predictability of the observed expert plays an important role. In this study, the expert is assumed to exhibit a high degree of consistency (e.g., a domain expert such as a climatology professor). This is reflected in an initially high trust value (β = 0.7), which is encoded in the strength of neural connections. Although trust may arise from both emotional and rational sources, the present model focuses on its rational component. This value is dynamically updated during the simulation based on observational learning.

The chosen case study revolves around transportation, offering choices such as car, bike, and public transport. In line with our study’s premise, the observer witnesses the expert’s decisions concerning these transportation modes and their outcomes. With this assumption, the observer tends to lean towards emotional decision making. Consequently, the initial preference for different transportation options is parameterized in terms of their emotional and rational valuation.

These values are purely model-based and do not imply any objective or normative classification of the options themselves. Rather, the distinction between “emotional” and “rational” reflects internal parameterization.

Finally, the model behaves deterministically when the initial conditions and parameter settings are fixed. No stochastic noise is included unless explicitly introduced. In cases where random initialization is required (for example, for synaptic weights), reproducibility is maintained by fixing the random seed, ensuring that all simulation results can be consistently replicated.

### 3.2. Neural Representation of Trust

The excitability of the neural units depends on the strength of the neural connections, which is influenced by intra-neural stimulation. Strong neural connections are capable of creating more accurate and coordinated neural patterns, resulting in simultaneous oscillations with high energy. The influence of observational learning on an individual’s decision making process manifests in the neural connection weights and neural excitability.

**Figure 6:**
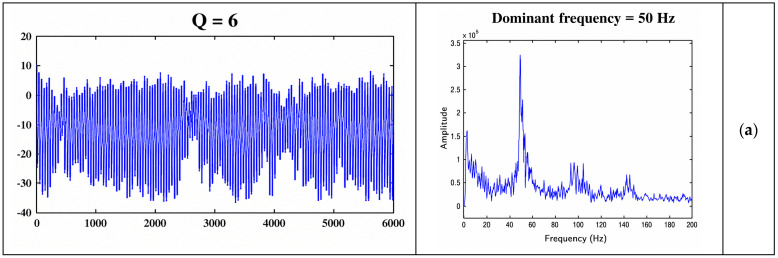

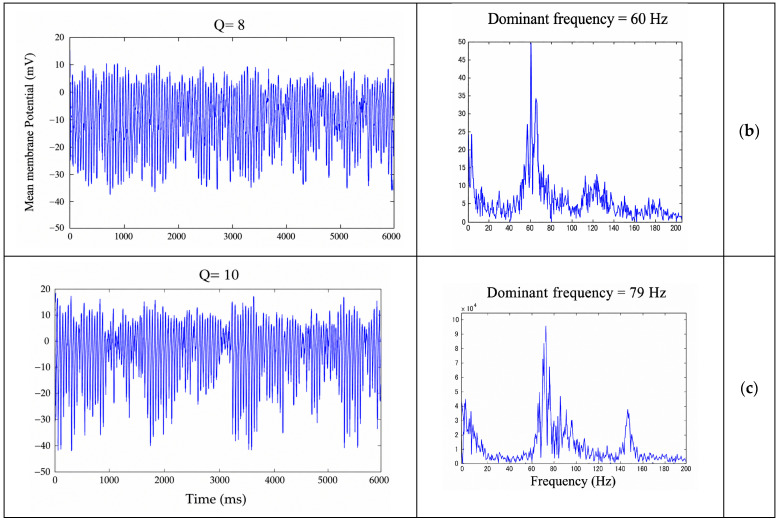
Oscillatory activities of the LPFC’s neural pattern corresponding to the public transport. The model shows that an increase in the variable representing trust leads to higher rational neural excitability and increased rational motivation. The higher the motivation becomes, the more regular and higher frequency the neural activity will be.

The oscillatory properties of neural patterns associated with the expert reflect the level of trust. Figure 6 illustrates the changes in LPFC’s oscillatory activities during observational learning. These results suggest that increases in trust lead to stronger and more coherent neural activity patterns within the model.

The neural arousal variable, Q, indicates the observer’s motivation to follow the expert’s actions and contributes to the excitability of neural units. Thus, increases in Q reflect a stronger neural encoding of trust-related motivation rather than constituting trust itself. Additionally, the dominant frequency of model-generated neural activity reflects signal energy, with higher frequencies corresponding to stronger neural responses. For instance, in Figure 6a, the dominant frequency of the corresponding neural pattern for expert 1 (trust level) is 50 Hz, with a Q value of 6. Increasing the Q value to 8 in Figure 6b and 10 in Figure 6c results in dominant frequencies of 60 and 79, respectively, indicating higher levels of trust.

Next, we explore the behavioral change in the observer from two different perspectives: (1) the impact of observational learning on trust and (2) the impact of trust on observational learning.

### 3.3. Impact of Observational Learning on Trust

The predictive signal representing the observer’s decision is proportional to the contingency of the action–outcome association. An expert with a high level of predictability (both in action and outcome) positively influences the rational trust of the observer. The level of trust in the expert is measured based on the oscillatory activities of the LPFC’s neural patterns. According to Equation (8), a high value of DBP results in high signal energy and an excitable neural pattern. The excitability of the corresponding neural pattern is computed as a function of motivation, Q, and the neural connection weights. Changes in the neural properties demonstrate the learning process leading to behavioral changes in the observer.

**Figure 7:**
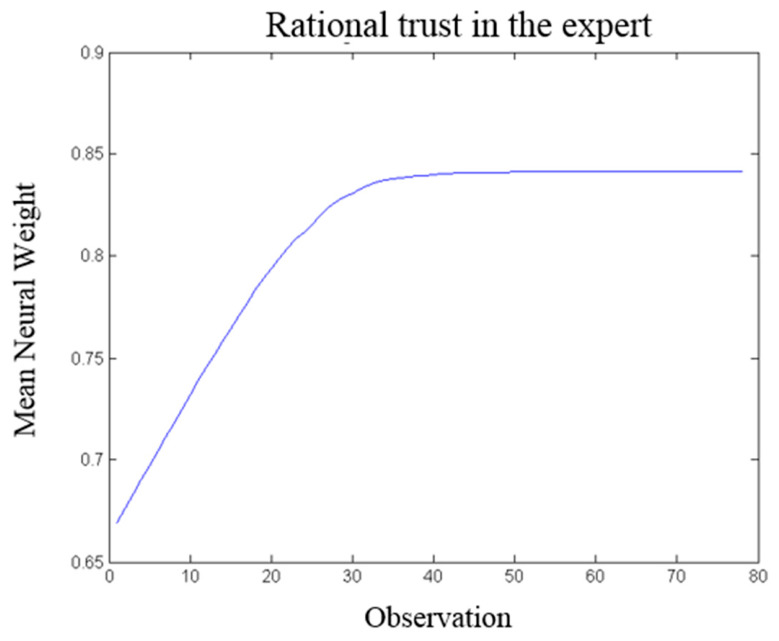
Illustration of the trust changes through observing the action-outcome of an expert. After 40 iterations, the level of trust reaches the steady-state value.

Figure 7 shows the trust learning curve. The changes in the connection weights during the observational learning process result from changes in the Q value, representing different levels of trust in the expert. The rate of change in the neural connection weights decreases during the observation of the expert, and the connections will eventually saturate, determining the stability of the observer’s trust in the expert. The increase in the strength of neural connections demonstrates an increase in the excitability of the cell assembly associated with the observed individual.

An increase in the trust level leads to enhanced neural representations of both emotional and rational values associated with the observed action. In the model, these values are reflected in the strength of synaptic connections between neural populations in the OFC and LPFC, which encode emotional and rational valuation, respectively, as described below.

### 3.4. Impact of Trust on Observational Learning

The attitude of the observer towards public transport (the expert’s action) is influenced by trust in the expert. In this part, we study the changes in the observer’s emotional and rational valuation according to changes in trust.

The strength of the rational signals projected by the LPFC’s cell assembly, corresponding to the expert, reflects the level of rational trust. The observer’s preference for the car suggests that the OFC signal for the car is stronger than that for public transport.

**Figure 8:**
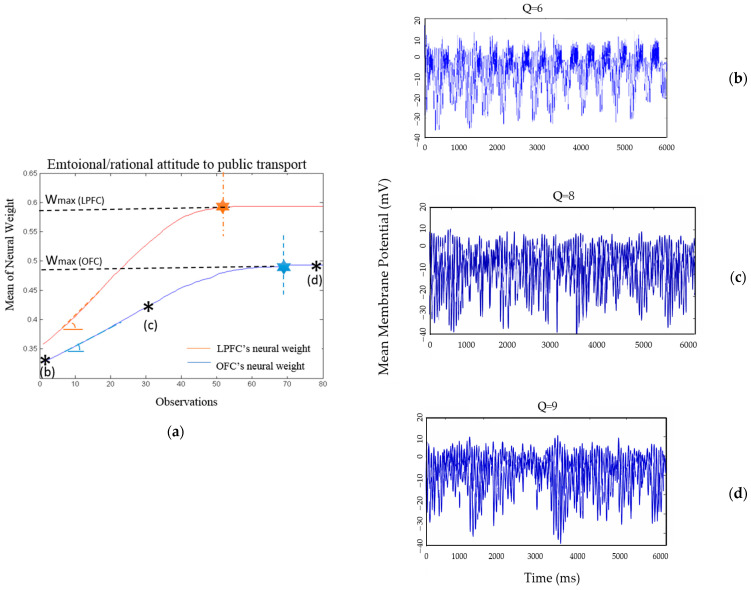
Illustration of the emotional and rational changes in the neural connection weights corresponding to the public transport pattern. The asterisk (*) marks the observation at which the system enters a steady state, characterized by zero learning dynamics and constant neural weights. (**a**). As an assumption, the emotional tendency to select public transport is less than the rational propensity. Observing an expert selecting the car with a successful outcome augments the rational valuation of public transport, albeit with a lower rate, and diminishes the emotional value. The oscillatory activities of OFC’s neural units associated with public transport are illustrated in (**b**–**d**). The emotional Q values increase, influenced by the LPFC’s modifications.

However, the emotional neural pattern linked to public transport exhibits lower excitability and weaker connection weights compared to its rational neural counterparts. As depicted in Figure 8, the observer initially demonstrates a higher rational inclination towards selecting public transport than its emotional value. As shown in Figure 7, an increase in rational trust positively influences how the observer evaluates the situation. As a result, the observer assigns a higher rational value to the expert’s action (i.e., public transport).

The increased simulated oscillatory activity of the LPFC neural pattern influences the oscillatory rhythms of the OFC neural pattern associated with public transport. Hence, the strength of OFC’s cell assembly corresponding to public transport increases, but at a lower rate compared to the increase in the LPFC’s neural pattern. Figure 8b–d illustrate the changes in the model-generated oscillatory activity of the LPFC’s cell assembly associated with public transport during the observational learning process. The emotional gain parameter,$Q_{emo}$ in the first frame is 6, and the neural weight connection will stabilize when the *Q* value approaches 9. Thus, the rational neural connection weights become saturated (Figure 8a, orange dotted line) before the emotional neural pattern reaches its maximum neural strength (Figure 8a, blue dotted line). Additionally, the maximum weight of the LPFC’s neural pattern is considerably greater than the saturation level of the OFC’s corresponding pattern.

In the following section, the properties of the emotional and rational cell assemblies associated with public transport during the observation of the expert’s actions and outcomes are measured. The excitabilities of the OFC’s and LPFC’s cell assemblies before the decision maker starts observing the action and outcome of the expert are as follows:

$${\vec{V}}_{Emo.,t=1}=\left[Car,Public\;transport,Bike\right]=\left[0.2,0.1,0.08\right]$$

$${\vec{V}}_{Rat.,t=1}=\left[0.1,0.2,0.05\right]$$

$${\vec{V}}_{final.,t=1}=[0.4,\;0.3,\;0.1]$$

Here, we assume the decision maker is a more emotional than rational person. Initially, she is more inclined to choose the car. However, her rational preference is to take public transport. In line with our assumption, the initial emotional value of public transport is less than its rational value. Hence, the mean neural weight of the emotional pattern associated with public transport is less than the rational neural weight of this option. The strength of the cell assembly corresponding to public transport after observing the action-outcome association of the expert is measured as follows:

$${\vec{V}}_{Emo.,t=30}=\left[Car,Public\;transport,Bike\right]=\left[0.223,0.256,0.089\right]$$

$${\vec{V}}_{Rat.,t=30}=\left[0.15,0.332,0.051\right]$$

$${\vec{V}}_{tot.,t=30}=\left[0.373,0.588,0.19\right]$$

Making a comparison between Figure 7 and Figure 8, the trust learning rate differs from the rate of change in emotional/rational reasoning. The results illustrate that the learning rate of trust in expert 1 is higher than the rate of emotional/rational learning about the expert’s action (i.e., public transport). Therefore, the required time for attitude change (towards public transport) is longer (almost twice as long) than the required time to form the attitude towards the expert.

## 4. Discussion

In this paper, we have presented our neurocomputational model, exploring the interplay between trust and observational learning, as well as the impact of social influence on an individual’s decisions. Our model is an attempt to demonstrate possible interactions between three cortical structures: OFC, LPFC, and ACC in a social-based decision making context. The involvement of OFC and LPFC in individual and social cognitive processes, as well as their interaction with ACC as a social hub, provides the framework for our model. The emotional and rational analyses of personal attitude and social information are the major contributions of OFC and LPFC, respectively, in this model. The conceptual grounding of this model is based on observational action and outcome prediction error signals recorded (as simulated) in OFC and LPFC, respectively. At the neural level, learning is defined based on the difference between the predicted and actual values in individual and social contexts. Hence, in this model, we take advantage of the observational prediction error signals to provide a basis for the observer to: (1) learn about and from the observed expert and (2) modify individual decisions and probably attitudes in the long term. Concerning the gathered knowledge about the expert, the observer makes an association between the observed action-outcome pair and the expert, which results in an increase/decrease in trust.

This model illustrates potential decision patterns of an observer in an observation-based decision making process. It also gains an understanding of the interplay between trust and observational learning. The model outcomes are based on the analyses of cell assembly activities (signal intensities and neural excitability).

The illustrated results can be analyzed from both neural and behavioral perspectives. As shown in Figure 6, there is a direct relationship between the properties of cell assemblies and the level of trust. A higher level of trust is reflected in stronger neural connection weights and higher neural excitability, resulting in less chaotic behavior. The simulated data illustrate how the oscillatory activities of neural patterns project the expectancy signal of option values. The higher the motivation becomes, the more regular the oscillatory activity will be.

Figure 8 depicts the oscillatory neurodynamics of both rational and emotional responses concerning the predictability of actions and outcomes resulting from the expert’s actions. Based on the simulation results, changes in emotional and rational values do not occur simultaneously. Rational value adjustments, influenced by rational trust, manifest at a faster rate compared to emotional changes. The stabilization of simulated rational oscillatory activity is observed sooner than that of the emotional counterpart. Despite the generally faster nature of emotional reasoning, as noted by Kahneman (2011) [36], we conclude that alterations in emotional preferences or attitudes require more time than changes in rational attitudes.

In summary, observing the actions and outcomes of others serves as a robust basis for both learning from and about them. Individual learning about others, driven by the predictability of their action-outcome associations, underpins the development of trust. Trust, along with the knowledge acquired from others, significantly shapes both emotional and rational valuation processes. Consequently, observational learning and trust emerge as two pivotal social variables profoundly influencing individuals’ behaviors and attitudes.

### Limitations

This work has some limitations that naturally point to directions for future development and continuation.

One important limitation concerns how cortical structures are represented. The orbitofrontal cortex, lateral prefrontal cortex, and anterior cingulate cortex are all modeled using a similar simplified three-layer neural architecture. While this choice ensures a computationally efficient framework, it should be noted that detailed anatomical differences are not considered. As a result, the model should be viewed as a conceptual account of large-scale interactions between cortical systems rather than a detailed neurobiological reconstruction. It reflects the general principles of interaction, but not the full biological complexity. Future work could address this by incorporating region-specific architectures and more realistic neural dynamics.

A second limitation is the absence of direct empirical validation. While the model produces internally consistent patterns of neural activity and behavioral adaptation, these results should be interpreted as illustrative rather than predictive. In particular, the EEG-like oscillatory patterns generated by the model represent abstract signatures of underlying processes. An important next step will be to quantitatively compare the model’s predictions with empirical behavioral and neurophysiological data.

Taken together, these limitations highlight both the current scope of the model and clear opportunities for refining and extending it in future work.

## 5. Conclusions

In this work, we introduced a phenomenological neurocomputational framework to examine how trust shapes observation-based decision making. By modeling the interaction between OFC, LPFC, and ACC, the study highlights trust as a central variable that links observational learning to changes in both neural dynamics and behavior. The results suggest that trust emerges from the predictability of observed action–outcome associations and influences how new information is integrated over time. In this sense, trust is not merely an outcome of learning but a driving factor in the evolution of emotional and rational valuation processes.

More broadly, the model provides a clear way to understand how trust influences learning and decision making in social contexts. It also highlights the importance of treating trust as a dynamic factor in explaining how individuals adjust their behavior in response to others.

## Figures and Tables

**Table 1:** The parameters chosen for the simulation of the three-layered networks in each of the three structures, ACC, OFC and LPFC. Many of these numbers are within physiologically realistic ranges.

N	Total Number of Network Units	450
$N_{ex}$	Number of excitatory neurons in each of the three networks	100
$N_{in}$	Number of inhibitory neurons in each layer	25
$\tau_{I}$	Decay time constant for feedforward inhibitory units	70 ms
$\tau_{II}$	Decay time constant for excitatory units	10 ms
$\tau_{III}$	Decay time constant for feedback inhibitory units	7 ms
$\lambda_{ex-ex}$	Space constant for excitatory-excitatory connections	5 mm
$\lambda_{ex-in}$	Space constant for excitatory-inhibitory connections	5 mm
d	Distance between nearest neighbors	1 mm
$t_{syn}$	Synaptic delays at all connections	0.8 ms
$v_{ex}$	Signal velocity in the excitatory-excitatory connection	0.5 m/s
$Q_{em}$	Emotional gain parameter	10
$Q_{rat}$	Rational gain parameter	6

## Data Availability

The original contributions presented in this study are included in the article. Further inquiries can be directed to the corresponding author.
